# Methanolic extract and nanosilver of *Feijoa sellowiana* reduce *Salmonella typhimurium* infection in female BALB/c mice

**DOI:** 10.1038/s41598-025-16334-5

**Published:** 2025-08-29

**Authors:** Sajad Batyari, Mohammad Ali Ebrahimzadeh, Mohammad Ahanjan, Mona Moshiri, Shadi Aghamohammad, Mehrdad Gholami

**Affiliations:** 1 Department of Medical Microbiology and Virology, Faculty of Medicine, Mazandaran University of Medical Sciences, Sari, Iran; 2 Pharmaceutical Sciences Research Center, School of Pharmacy, Mazandaran University of Medical Sciences, Sari, Iran; 3 Department Immunology, Faculty of Medicine, Mazandaran University of Medical Sciences, Sari, Iran; 4 Department of Bacteriology, Pasteur Institute of Iran, Tehran, Iran; 5 Molecular and Cell Biology Research Center, Faculty of Medicine, Mazandaran University of Medical Sciences, Sari, Iran

**Keywords:** *Salmonella*, *Feijoa* fruit, BALB/c mice, Nanosilver, Antibacterial activity, Microbiology, Diseases

## Abstract

Increasing resistance to antibiotics has led to research into new treatment options. Recent studies suggest the use of plant extracts as an alternative strategy. *Feijoa* is widely known for its efficacy against a broad spectrum of bacterial pathogens. In this study, the antibacterial activity of methanolic extract and nanosilver of *Feijoa* fruit (FF-AgNPs) was investigated in BALB/c mice infected with *Salmonella enterica* serovar Typhimurium (*Se*sT). The antimicrobial effect of *Feijoa* fruit and FF-AgNPs was evaluated by employing minimum inhibitory concentration (MIC) and minimum bactericidal concentration (MBC) against *Se*sT. The efficacy of the treatment in vivo was evaluated in mice by quantifying the viable population of *Ses*T ATCC 14028 purchased from the Iranian Biological Resource Center. Both MIC and MBC results showed a strong bactericidal effect of the *Feijoa* fruit and its nanosilver against *Se*sT. According to the colony count and weight measurement results of the mice, methanolic extract of *Feijoa* fruit and FF-AgNPs had acceptable antimicrobial efficacy. Also, none of the mice receiving these agents died, while a high mortality rate was observed in the infected group. Due to the continuous increase in microbial resistance and the importance of pathogenic bacteria in the healthcare system, there is a growing need for the use of complementary agents that possess antimicrobial properties. The findings of the current study suggest that methanolic extract of *Feijoa* fruit and FF-AgNPs can be considered as a beneficial antibiotic supplements due to their favorable antimicrobial effects.

## Introduction

The range of illnesses caused by food poisoning ranges from mild, self-limiting gastroenteritis to severe, life-threatening complications. Studies show that there are approximately 38 million cases of food poisoning in the United States each year^1^. Between 1996 and 2011, a total of over 600,000 cases of *Salmonella* isolates were documented in the human population which corresponds to an average annual incidence rate of 13.1 cases per 100,000 people^2^.

*Salmonella* is a gammaproteobacterial bacterium from the *Enterobacteriaceae* family. *Salmonella enterica* is of clinical importance as an infectious agent in the human population. *Salmonella enterica* Serovar Typhimurium, a Gram-negative pathogen, plays a critical role in causing foodborne diseases and gastroenteritis in millions of individuals worldwide annually^3^. 93.8 million cases of gastroenteritis^4^, 11–22 million cases of typhoid and paratyphoid^5^, 155,000 deaths occur worldwide^6^ as a result of *Salmonella* infection. Since animals can serve as reservoirs for *Salmonella* for transmission from animals to humans is possible, it is imperative to develop treatment strategies for this bacterium in conjunction with the high prevalence of *Salmonella* infections (as shown by the prevalence of salmonellosis in food)^7^.

Fluoroquinolones, antibiotics with a broad spectrum of activity, third generation of cephalosporins (specifically ceftriaxone), and in some cases, tetracycline are frequently used for the treatment of *Salmonella* infections. The emergence of resistance to these antibiotics has recently become a significant public problem^8^. The emergence of antibiotic resistance, which includes bacteria with multiple drug resistance (MDR), is attributed to a number of mechanisms of antibiotic resistance, in particular the horizontal transmission of resistance genes in bacteria^9^. The emergence of antibiotic resistance, which is considered as the most significant evolutionary alteration in contemporary society, has led to a decisive increase in nosocomial infections, longer periods of illness and increased costs associated with hospitalization^10^.

Increasing resistance to antibiotics has led to research into new treatment options. A recent study suggests the use of plant extracts as an alternative strategy^11^. *Feijoa*, scientifically known as *Acca sellowiana*, belongs to the Myrtaceae family and is mainly cultivated in tropical and subtropical regions. These regions include southern Brazil, Uruguay, Paraguay, and northern Argentina^12^. Its fruit is a natural source of valuable bioactive compounds such as phenolic compounds^13^. A very good protective effect against hypoxia in all the hypoxic models have reported for *Feijoa* Fruit extract^14^. In addition, *Feijoa* fruit extract is a valuable source of antioxidant, anti-inflammatory and antinociceptive activities^15^. The aqueous extract of *Feijoa* fruit has shown both antibacterial and antioxidant properties as determined by the quantitative luminescence method^16^.

As said above, the use of alternative antimicrobial agents, such as plants, has become noticeable in recent years. In addition, the use of nano-carriers or nano-antimicrobials has gained attention as a strategy. This is due to their inherent antimicrobial activity, such as in nanoparticles, or their ability to improve the overall efficacy of entrapped antibacterial agents^17^. Nano-drugs, which have only recently entered the field of therapeutic strategies, have the potential to make a remarkable contribution in various areas, particularly in the diagnosis and treatment of malignancies, infectious diseases and neurological disorders. In addition to combating antimicrobial resistance, these agents offer alternative and more efficient approaches that can lead to significant advances in the above scenarios^18–20^.

Various metals such as copper, magnesium, gold, zinc, titanium and silver were investigated with regard to their use as nanoparticles^21^. Green synthesis of silver nanoparticles from *Feijoa* fruit has been reported recently by our team. These nanoparticles displayed powerful anticancer activities against two tumor cell lines with little effect on BEAS-2B normal cells^22^. However, a more detailed evaluation of the above-mentioned beneficial properties in a mouse model could provide a more comprehensive demonstration of their efficacy. In the present study, we aimed to evaluate the antibacterial activity of the methanolic extract and nanosilver of *Feijoa* fruit in BALB/c mice infected with *Se*sT.

## Methods

### The isolation and identification of *Salmonella* species

*Salmonella enterica* serovar Typhimurium (*Se*sT) ATCC (14028) was purchased from the Iranian Biological Resource Center (IBRC) Microorganisms Bank. According to the center’s guidelines, the bacteria were cultured on tryptic soy agar (TSA) medium under aseptic conditions using a biosafety cabinet class II. The TSA medium was freshly prepared and pre-warmed. Environmental parameters such as temperature (37 ± 0.5 °C) and humidity were controlled and monitored, and then incubated at 37 °C for 24 h. The bacteria were identified by biochemical tests such as SIM, TSI, LIA, MRVP, Urea, Simmons citrate, and ONPG. The results of the biochemical tests were checked using the API kit (bioMérieux, France, catalog number: 1007181060) according to the manufacturer’s instructions.

### The preparation of *Feijoa sellowiana* fruit extract and FF-AgNPs

The *Feijoa* fruit extract was prepared following a standardized maceration procedure, as described in our previous study. *Feijoa* fruit was collected from the Fajr Citrus Experimental Institute. FF-AgNPs were freshly suspended with ultrasonication at 37 °C for 15 min before administration, and *Feijoa* extract was prepared in sterile distilled water at 100 mg/mL concentration. In October–November 2017, the plant was identified by Dr. Bahman Eslami, and a voucher specimen (No. 194) was deposited in the herbarium of the Sari School of Pharmacy. The fruit was dried at room temperature and ground before extraction. One hundred grams of powder was extracted at room temperature (RT) using the maceration method, with methanol as the extracting solvent. The extracts were separated from the residue by filtration using filter paper. The resulting extracts were concentrated in a vacuum using a rotary evaporator at 35°C until crude solid extracts were obtained. The crude extracts were then freeze-dried to ensure complete dryness, yielding 31.5 g of extract^22^. In our previous study, the preparation of FF-AgNPs has also been performed and described in detail^22^.

### HPLC analysis

The phenolic compounds present in the *Feijoa* fruit extract were analyzed using High-Performance Liquid Chromatography (HPLC) as described previously by our team^23^. The HPLC system employed a K-1001 solvent delivery unit, a Rheodyne injection valve with a 20 µL sample loop, and a UV–vis detector (model K-2600) set to 254 nm, all from Knauer Assoc., Germany. Separation was achieved using an ODS-C18 column (250 mm × 4.6 mm I.D., 5 µm particle size, Shim-pack VPODS). Prior to analysis, all solvents were filtered and degassed. A binary solvent system, consisting of solvent A (H_2_O with 10% AcOH) and solvent B (CH3CN) in a 95:5 ratio, was used. The flow rate was maintained at 1 mL/min, and all measurements were performed at ambient temperature.

### In vitro antimicrobial activity assay in *Feijoa* fruit extract, FF-AgNPs and ceftriaxone

#### Minimum inhibitory concentration (MIC)

The MIC tests were determined in 96-well plates (Costar; Corning Incorporated, Corning, NY, USA) based on a modified methodology of the National Committee for Clinical Laboratory Standards. Briefly, broth dilution method was performed for antibacterial tests by preparing serial dilution of FF-AgNPs, *Feijoa* fruit extract and ceftriaxone in a Mueller Hinton Broth (MHB) medium (Merck, Germany). 100 μL of *Feijoa* fruit extract (with an initial dilution of 100 mg/mL) and 100 μL of FF-AgNPs (with an initial dilution of 500 ppm) were incorporated on to microplates. Serial dilutions of 0.25–16 µg/mL were added to assess the efficacy of the 100 μL of the ceftriaxone. The negative control consisted of 100 μL MHB medium and 100 μL FF-AgNPs. The positive control consisted of 100 μL MHB and 100 μL of a bacterial suspension in 0.5 McFarland dilution. Finally, 100 μL of the *Se*sT strain with a dilution of 0.5 McFarland (with a dilution of 1/100) was added to the wells. The microplate was subsequently placed in a 37 °C incubator overnight. After a period of 24 h, the results were analyzed. The first case in which no visual turbidity was observed was considered MIC^22^. Quality control was performed using *Escherichia coli* ATCC 25922 as a reference strain, with MIC tests conducted in triplicate.

#### The minimum bactericidal concentration (MBC)

The MBC was determined following the MIC assay. From each well that showed no visible bacteria growth, 10 µL of the suspension was aseptically taken and spread onto Mueller–Hinton agar (MHA). The plates was then incubated at 37 °C for 18–24 h. The lowest concentration at which no bacterial colonies were observed was recorded as MBC^22^.

### In vivo antimicrobial activity assay

#### Animals and experimental design

The animal experiment protocol was approved by the Animal Experimentation Committee of Mazandaran University of Medical Sciences (IR.MAZUMS.REC.1400.9193). All methods were carried out in accordance with relevant guidelines and regulations and are reported in accordance with ARRIVE guidelines. A total of twenty-four female BALB/c mice, aged 6–8 weeks and weighing between 18 and 25 g, were obtained. All mice were maintained in a controlled environment with a 12-h light–dark cycle and at a controlled room temperature. All experimental groups had adequate access to water and food. After a 2 weeks acclimatisation period, mice were randomly divided into eight groups using a random number generator. The use of 3 mice per group was approved by the institutional ethics committee in accordance with national guidelines for pilot animal studies. All mice were housed in identical environmental conditions (22 ± 2 °C, 55 ± 5% humidity, 12-h light/dark cycle). The groups can be categorized as follows: (A) The positive control group consists of mice infected orally by gavage with *Ses*T at a cell density of 1.5 × 10^8^ CFU/mL without receiving any treatment, (B) The negative control group, consisting of mice that only receive water and food, (C) A group of mice administered FF-AgNPs, (D) A group of mice receiving *Feijoa* fruit extract, (E) A group of mice receiving the antibiotic ceftriaxone, (F) A group of mice infected with *Ses*T and subsequently treated with FF-AgNPs, (G) a group of mice infected with *Ses*T and subsequently treated with *Feijoa* fruit extract, (H) a group of mice infected with *Ses*T and subsequently treated with the antibiotic ceftriaxone.

Prior to conducting the experiments, the mice were administered water containing streptomycin at a concentration of 5 mg/ml to reduce the presence of facultative anaerobic bacteria that typically inhabit the gastrointestinal tract of mice as part of their normal microbial flora. Extracts were then prepared from *Feijoa* fruit containing both FF-AgNPs and ceftriaxone at concentrations equivalent to MBC. The sterile physiological extracts were dissolved in FF-AgNPs and ceftriaxone, respectively, using sterile distilled water as a solvent. On the first day (day zero), fecal samples were collected from all groups of mice, and the bacterial content (colony count) was measured.

On the second day, a solution of 1 mL of normal saline with a concentration of 1.5 × 10^8^ CFU/mL *Se*sT (at a McFarland value of 0.5) was prepared and orally administered to the eligible groups of mice. It is important to note that the negative control group of mice received 1 mL of normal saline to establish a standard for the experiment (called the gavage stress factor). One hour after *Salmonella* infection in the eligible groups, each group received their respective treatment regimen orally, which consisted of 1 mL of FF-AgNPs, 1 mL of *Feijoa* fruit extract, and an antibiotic at a concentration equivalent to the MBC. Fecal samples were taken from all groups of mice on the second day and the bacterial count was determined. Similarly, on the third to seventh day after the administration of the drug regimen to each group, fecal samples were collected and the bacterial counts were determined. On the seventh day, one mouse from each group was euthanized and the colon of the mice was removed for histopathological examination. However, the remaining mice in each group were kept alive until day 12, at which point all mice were euthanized. After the designated treatment timeframe, all mice were sacrificed by cervical dislocation under anesthesia using ketamine and xylazine along with atropine sulfate at dose 200/15 mg/kg and atropine 0.05 mg/kg. The combinations were diluted in sterile saline and 0.1 mL per 10 g of body weight was injected intraperitoneally.

#### Bacteriologic analyses

Fecal samples were collected aseptically from each mouse in all experimental groups. One gram of feces was weighed and homogenized in 1 mL of sterile saline solution. The suspension was then serially diluted in sterile saline, and 10 μL of each dilution was plated onto Xylose Lysine Deoxycholate (XLD) agar (Merck, Germany). The plates were incubated at 37 °C for 24 h. Colonies were initially identified based on their characteristic morphology on XLD agar—typically red colonies with black centers. To confirm the identity of the colonies, representative isolates were further subjected to biochemical testing using the API kit. The number of confirmed Salmonella colonies was recorded, and colony-forming units (CFU) per gram of feces were calculated accordingly^24^.

### Body weight

The change in body weight was determined using the equation (final BW—initial BW/initial BW) and the weight reduction was calculated.

#### Histopathologic examination

The tissue samples were placed in a ten percent formalin solution and then embedded in paraffin. Blocks of paraffin with a thickness of three microns were prepared and stained with hematoxylin and eosin (H&E). An independent pathologist then assessed the stained slides. Scanning and image processing of the slides was performed using ImageJ scanner and viewer software. The histopathological differences for each change in each of the studied groups were determined and scored by an independent observer who was blinded to the study. The colonic tissue sections were examined and scored for pathological findings, especially inflammation^25^.

#### Statistical analysis

The results were analyzed using Statistical Package for the Social Sciences (SPSS) version 24. A significance level of *p* < 0.05 was considered statistically significant. For group comparisons, normality of the data was checked using the Shapiro–Wilk test, followed by analysis of variance (ANOVA). Due to its control of Type I error, the Bonferroni test was chosen for post hoc pairwise comparisons. Given the significant differences among the groups, a post hoc Bonferroni pairwise comparison test was conducted.

## Results

### HPLC separation of phenol derivatives

**Fig. 1:**
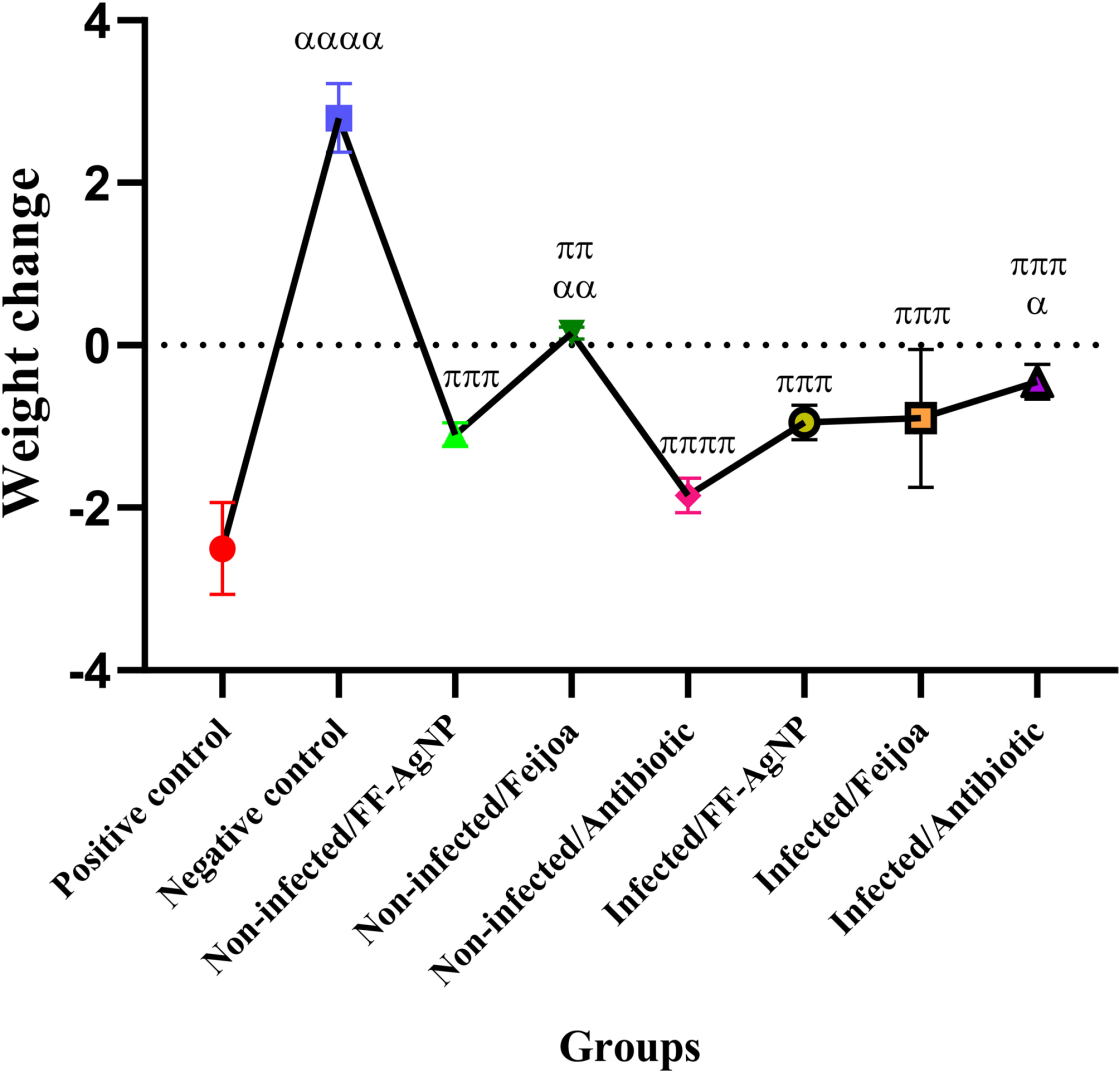
Effects of FF-AgNPs, *Feijoa* extract, and ceftriaxone on body weight changes. Data are presented as the mean ± SD, N = 3 per group. Statistical significance was determined using the following symbols: α, p < 0.05; αα, *p* < 0.01; ααα, *p* < 0.001; αααα, *p* < 0.0001 (Positive group vs. Other groups) and π, *p* < 0.05; ππ, *p* < 0.01; πππ, *p* < 0.001; ππππ, *p* < 0.0001; (Negative group vs. Other groups).

The major components were identified, including catechin (188.5 mg/g of extract), gallic acid (18.5 mg/g), caffeic acid (3.2 mg/g), rutin (15.8 mg/g), and p-coumaric acid (4.7 mg/g). The chromatogram and phenolic profiles have been previously published^22^, with detailed chromatographic data and retention times provided in Fig. 1 and Table 1 of mentioned study.

**Table 1:** Effect of treatment with *Feijoa* fruit extract, FF-AgNPs and ceftriaxone.

Days/groups	0	1	2	3	4	5	6	7	8	9	10	11	12
Positive control	0	2 × 10^3^	4 × 10^3^	3 × 10^3^	5 × 10^4^	4 × 10^3^	1* 4 × 10^3^	1* 2*	–	–	–	–	–
Negative control	0	0	0	0	0	0	0	0	0	0	0	0	0
Not-infected/FF-AgNPs	0	0	0	0	0	0	0	0	0	0	0	0	0
Not-infected/*Feijoa*	0	0	0	0	0	0	0	0	0	0	0	0	0
Not-infected/Antibiotic	0	0	0	0	0	0	0	0	0	0	0	0	0
Infected/FF-AgNPs	0	4 × 10^3^	4 × 10^5^	4 × 10^5^	3 × 10^3^	4 × 10^3^	4 × 10^3^	2* 3 × 10^3^	3 × 10^2^	3 × 10^2^	2 × 10^2^	2 × 10	2 × 10
Infected/*Feijoa*	0	3 × 10^3^	3 × 10^5^	4 × 10^3^	5 × 10^3^	3 × 10^3^	3 × 10^3^	2* 2 × 10^3^	3 × 10^3^	2 × 10^3^	2 × 10^3^	2 × 10^2^	2 × 10^2^
Infected/Antibiotic	0	3 × 10^2^	4 × 10^3^	3 × 10^3^	3 × 10^3^	2 × 10^2^	1 × 10^2^	2*	0	0	0	0	0

^1^*Natural death of mice.

^2^*Killing mice for histopathological examination.

### Determination of MIC and MBC

The *Feijoa* fruit extract, FF-AgNPs, and ceftriaxone displayed a MIC of 100 mg/L, 250 ppm, and 0.5 mg/L, respectively. Correspondingly, the MBC of the *Feijoa* fruit extract, FF-AgNPs, and ceftriaxone were 100 mg/L, 500 ppm, and 1 mg/L, respectively.

### In vivo antibacterial activity

#### Treatment efficacy on colony count

The number of bacterial colonies can be found in Table 1. In the first days after infection, all groups exposed to *Se*sT exhibited an increase in fecal bacterial load, indicating a successful infection. These findings demonstrated an increase in colony counts in the positive control mice, leading to the death of two mice on the sixth and seventh day, respectively. As expected, the groups that were not infected with *Se*sT showed no signs of the presence of bacteria in the feces. In the mice infected with *SesT* and subsequently treated with ceftriaxone, the bacterial load in the feces decreased and reached zero on the seventh day (*p* < 0.001, compared to the positive control group). Similarly, the groups treated with FF-AgNPs and *Feijoa* fruit extract exhibited a significant reduction in bacterial load compared to the positive control group (*p* < 0.01 and *p* < 0.05, respectively). However, the reduction in bacterial count was most significant in the FF-AgNP-treated group, with a marked decrease in bacterial load observed from day 8 onward (*p* < 0.01).

#### Body weight

The results related to body weight changes are presented in Fig. 1. Mice in the infected untreated group (*Ses*T) showed a significant decrease in body weight compared to the negative control group (healthy, uninfected mice). In contrast, healthy mice without any treatment exhibited a significant increase in body weight over the study period. Treatment with FF-AgNPs and the antibiotic in uninfected mice did not result in statistically significant weight changes, indicating that these treatments were well tolerated and did not adversely affect general health. Interestingly, administration of *Feijoa* fruit extract to infected mice significantly improved body weight compared to the infected untreated group (positive control), suggesting a potential protective or restorative effect. However, no statistically significant difference in weight reduction was observed among the infected mice treated with *Feijoa* fruit extract, FF-AgNPs, or the antibiotic, indicating comparable efficacy in mitigating infection-induced weight loss. To minimize the influence of external factors on body weight, all animals were maintained under identical environmental conditions (22 ± 2 °C temperature, 55 ± 5% humidity, and a 12-h light/dark cycle) and had free access to a standard commercial diet and water (ad libitum) throughout the experiment. Daily monitoring and consistent handling by the same personnel were performed to reduce stress and variability.

#### Survival rate

The survival status of mice was monitored daily across all experimental groups during the 12-day observation period. As shown in Table 1, two mice in the positive control group (infected with *Se*sT and receiving no treatment) died naturally on days 6 and 7. In contrast, all mice in the treatment groups—including those receiving *Feijoa* fruit extract, FF-AgNPs, or ceftriaxone—survived until the end of the experiment. While formal Kaplan–Meier survival analysis was not feasible due to the limited sample size per group (n = 3), the observed survival outcomes support the protective effect of the tested agents against systemic *Salmonella* infection in mice. These findings reinforce the microbial load reduction trends observed in the treated groups.

#### Histopathology

**Fig. 2:**
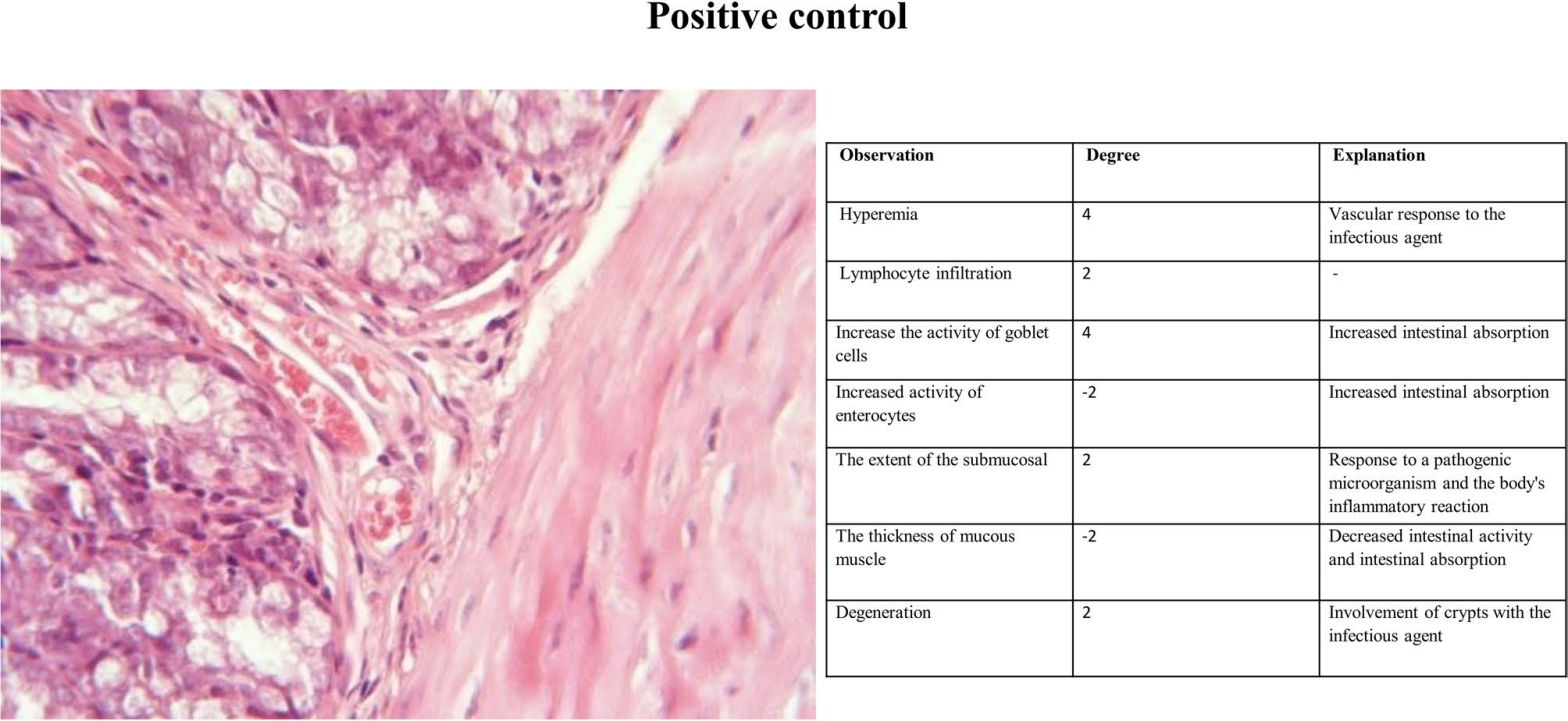
H&E staining of colonic tissue sections in positive control group.

**Fig. 3:**
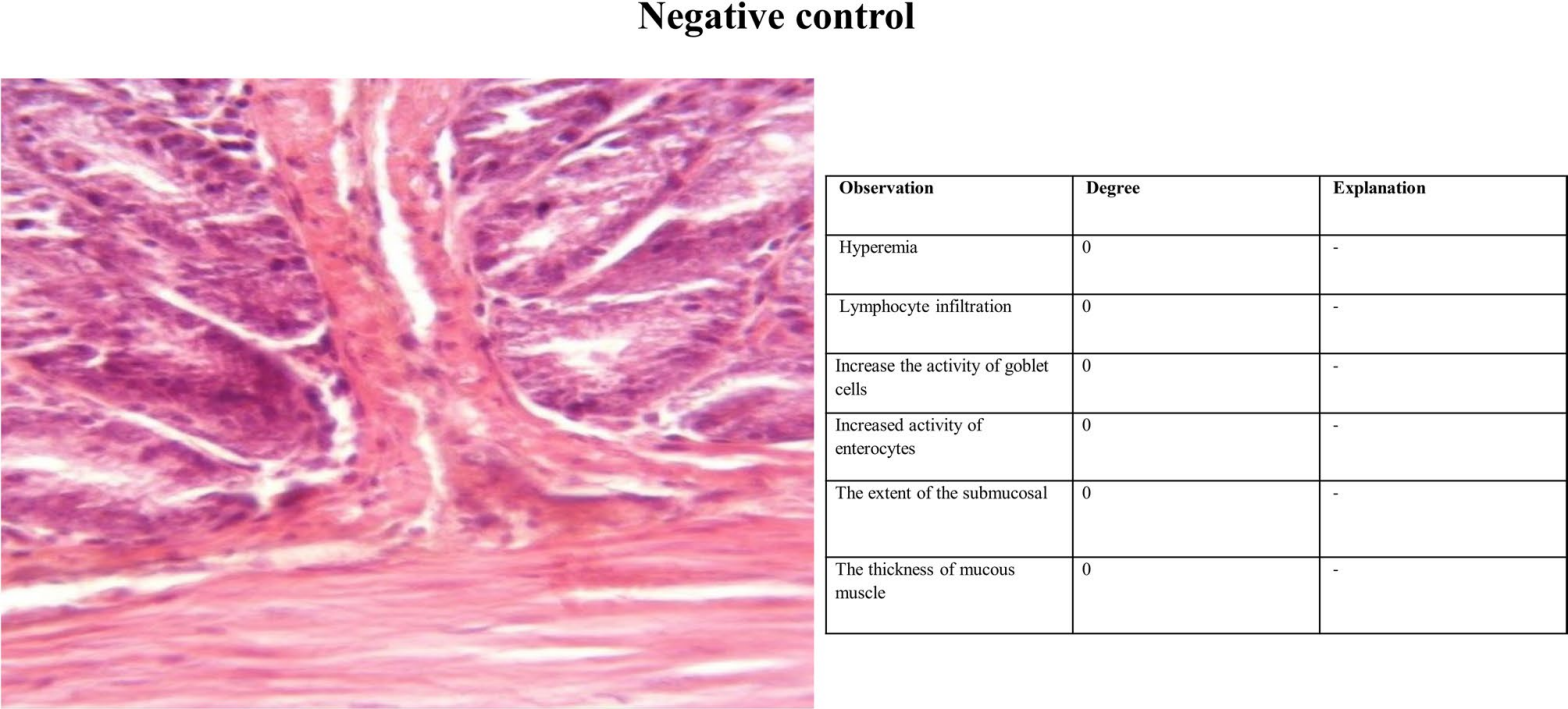
H&E staining of colonic tissue sections in negative control group.

**Fig. 4:**
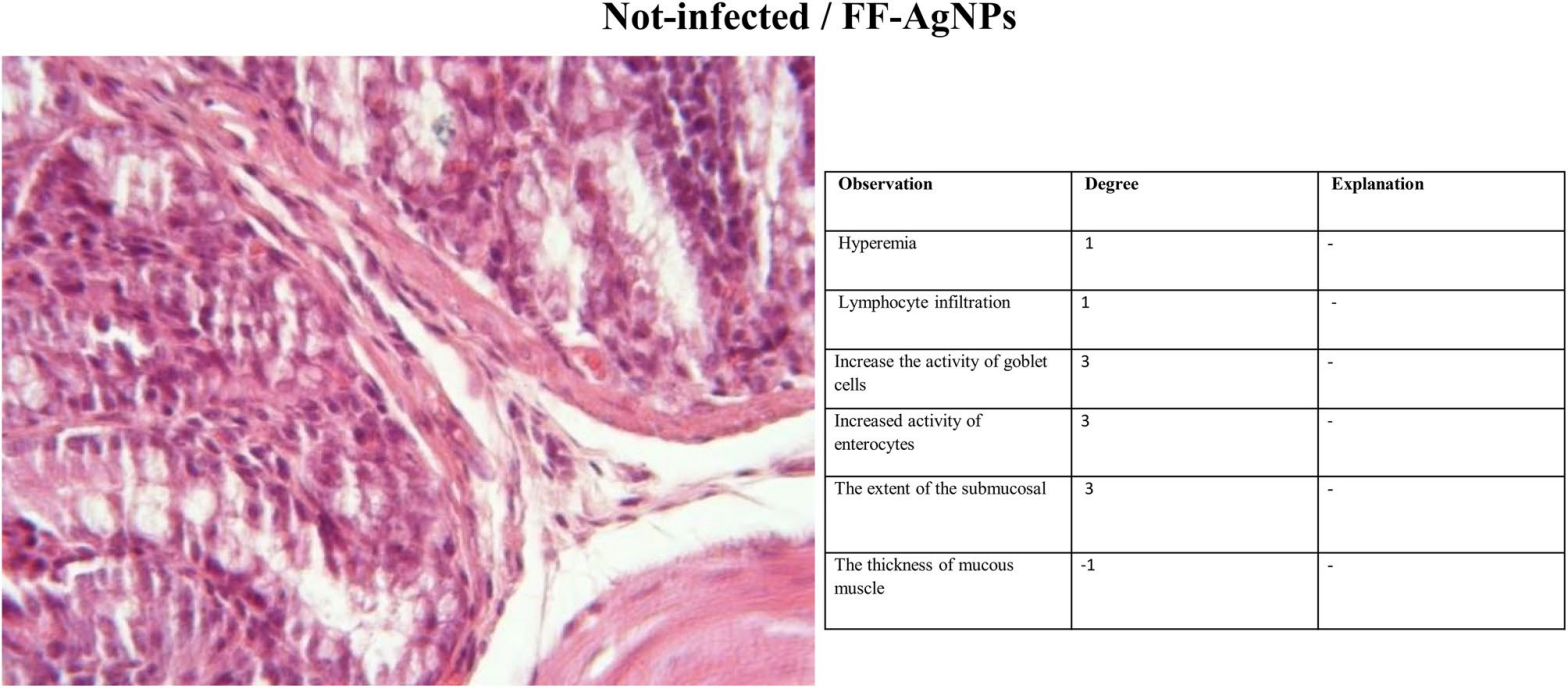
H&E staining of colonic tissue sections in not-infected/nano-treated group.

**Fig. 5:**
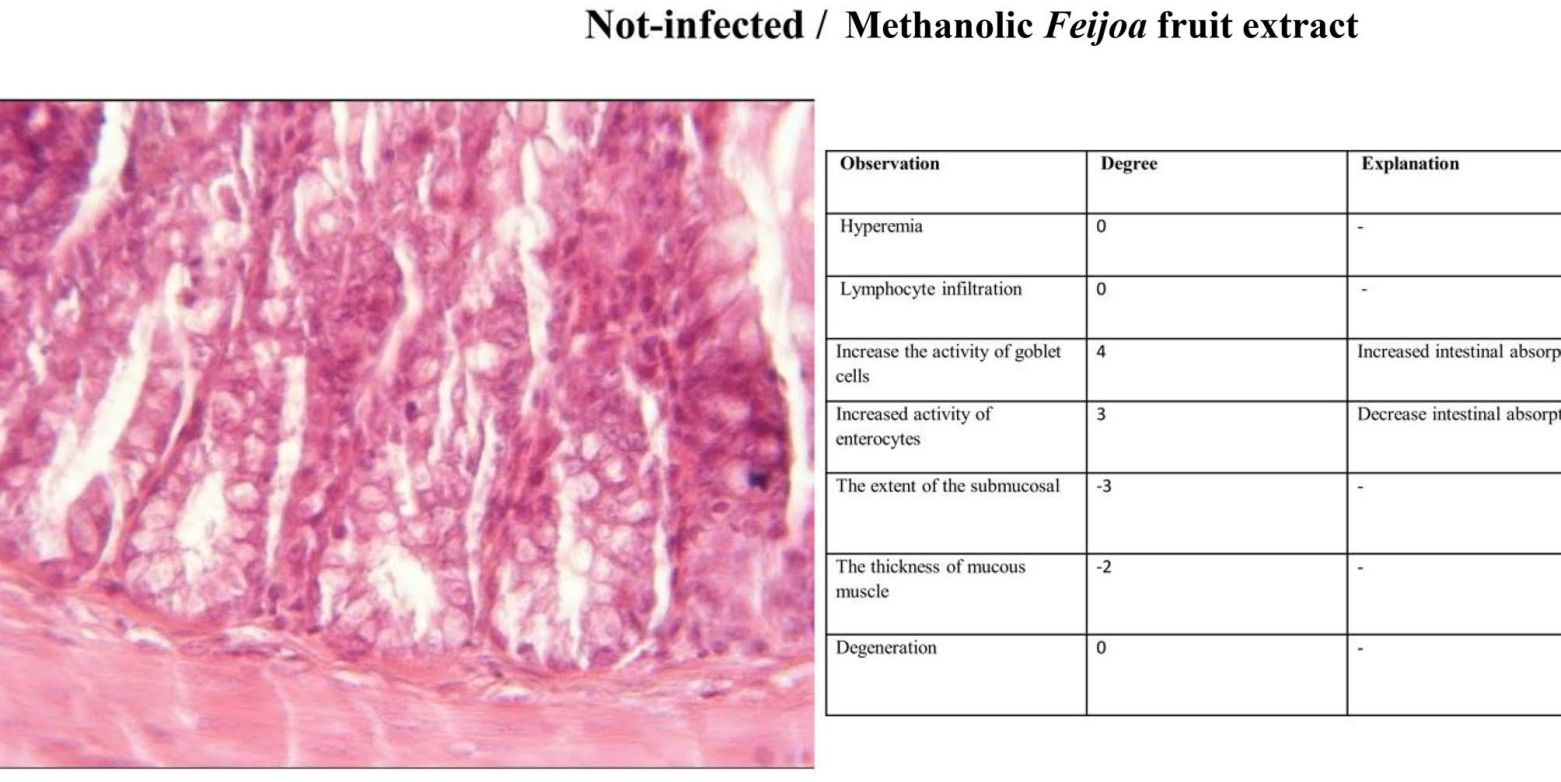
H&E staining of colonic tissue sections in not infected/*Feijoa* fruit extract-treated group.

**Fig. 6:**
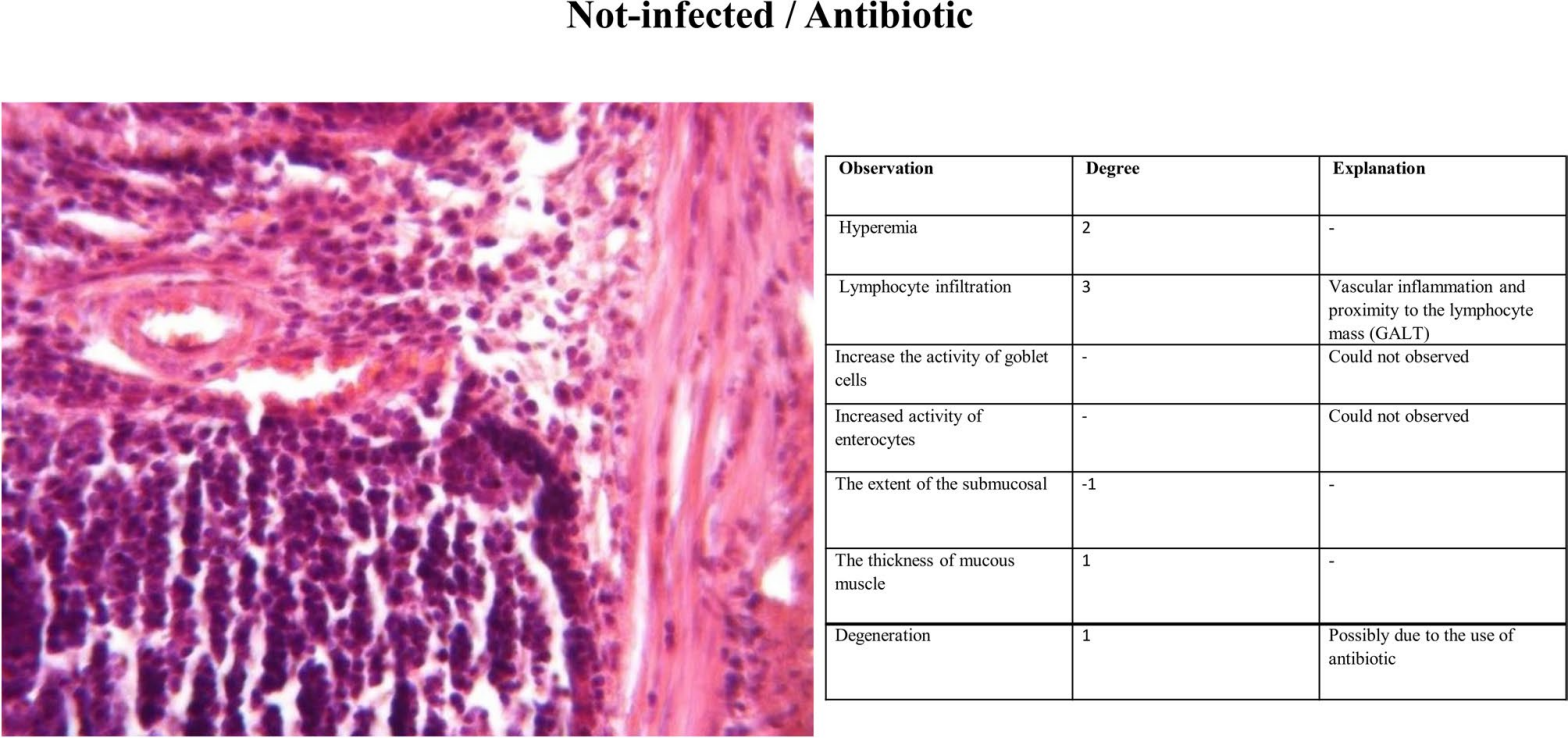
H&E staining of colonic tissue sections in not infected/antibiotic-treated group.

**Fig. 7:**
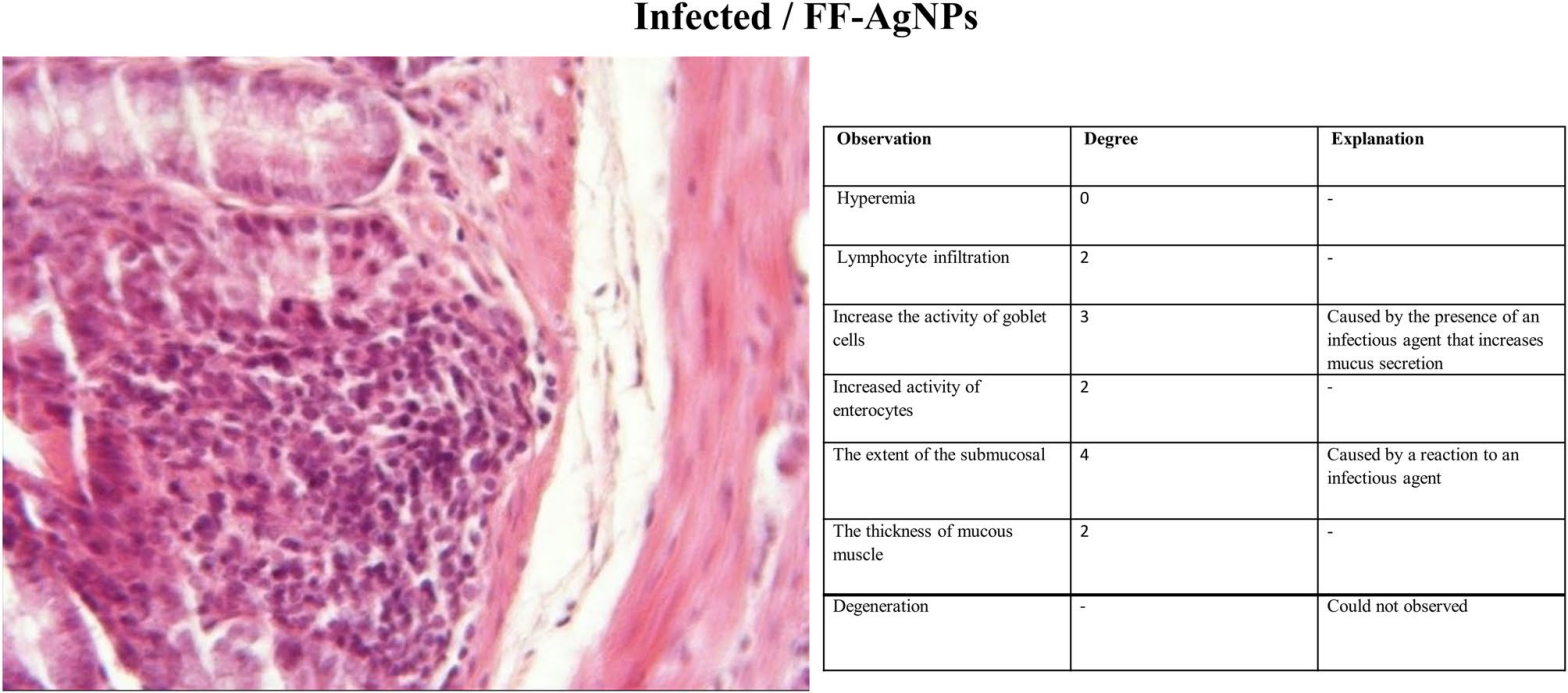
H&E staining of colonic tissue sections in infected/nano-treated group.

**Fig. 8:**
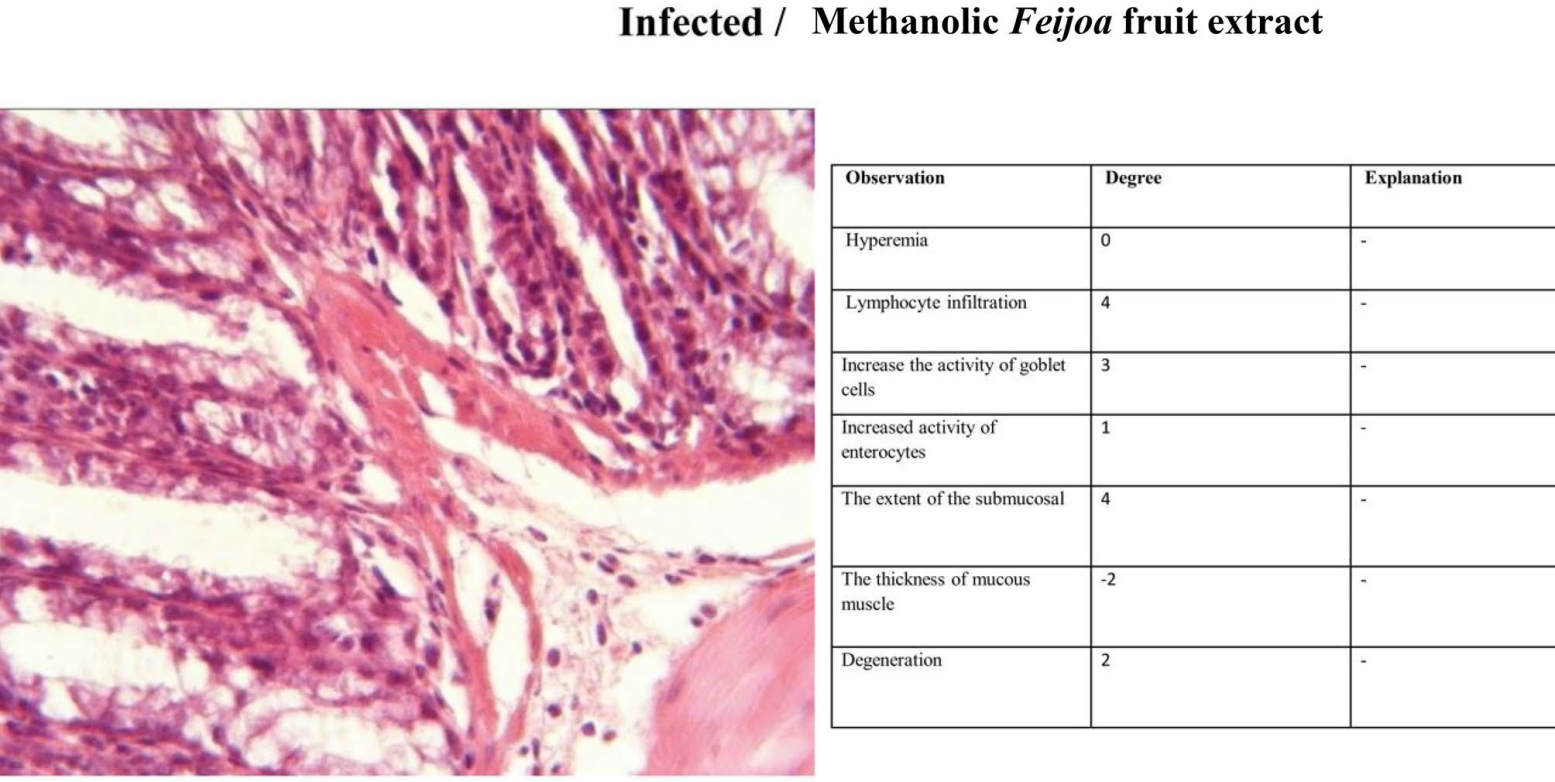
H&E staining of colonic tissue sections in infected/*Feijoa* fruit extract-treated group.

**Fig. 9:**
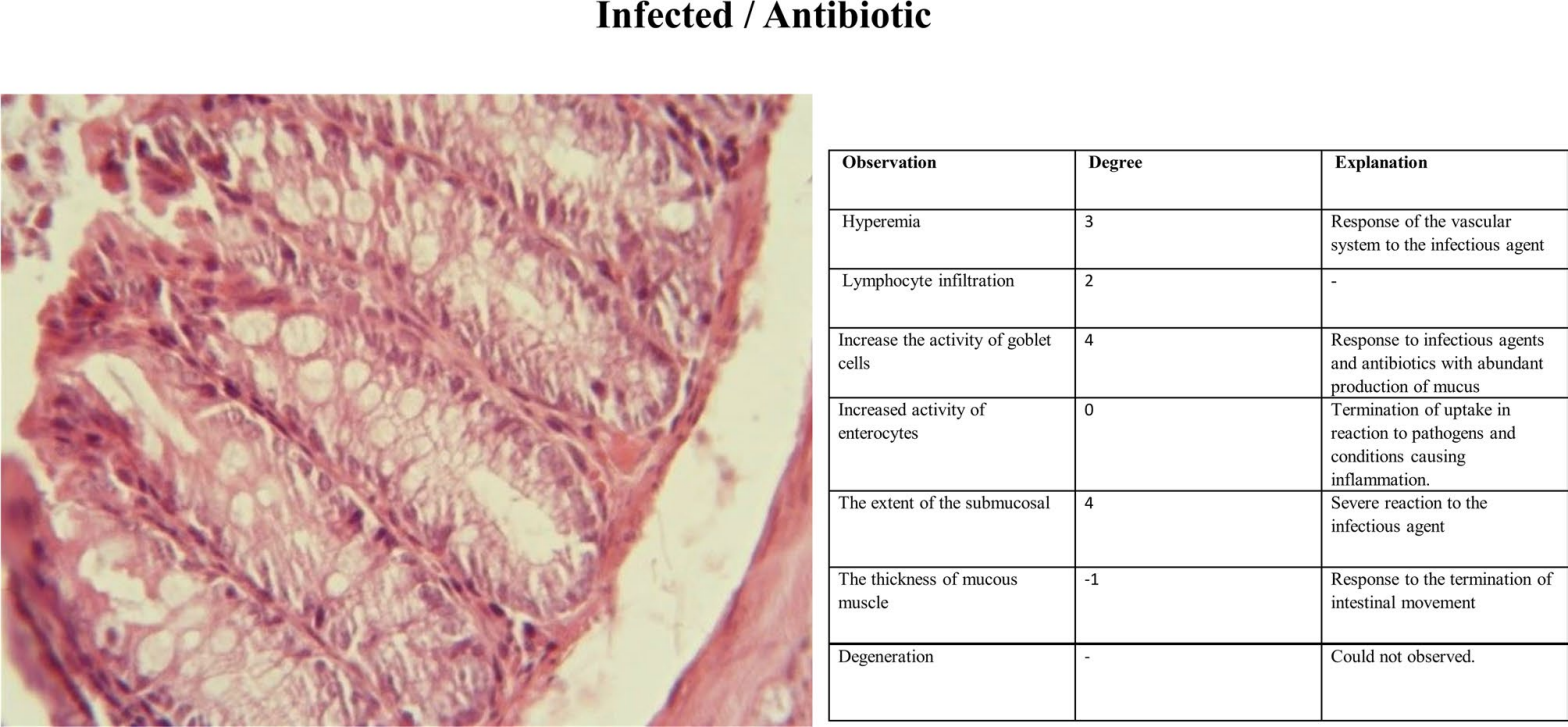
H&E staining of colonic tissue sections in infected/antibiotic-treated group.

The histopathological analysis was performed using H&E staining. The results are shown in Fig. 2, 3, 4, 5, 6, 7, 8 and 9. The histopathologic examinations were performed in accordance with the control groups and yielded the following results. In the positive control group, the colon showed considerable infiltration of lymphocytes with basophilic nuclei, alongside extensive hemorrhage and leakage of erythrocytes. More details could be seen in Fig. 2. Conversely, the negative control group exhibited no signs of localized damage or damage and showed a generally normal condition, Fig. 3.

The third group (Non-infected/FF-AgNPs) exhibited a mild degree of local inflammation, so it was not considered further, however, no signs of bleeding or vascular congestion were observed. Increased goblet cell and enterocyte activity was also observed in this group (Fig. 4). The fourth group (Non-infected/methanolic extract of *Feijoa* fruit), Fig. 5, displayed no lymphocytes and no inflammation within the examination site, and also no signs of erythrocyte leakage or other pathological lesions were present. In the fifth group (Non-infected/Antibiotic), significant infiltration of inflammatory cells was observed, and slight mucosal thickness and vascular degeneration could be seen in Fig. 6. In the sixth group (Infected/FF-AgNPs), condition of the tissue improved due to previously moderate inflammation, with no more red blood cells visible. Goblet cell and enterocyte activity was also seen in Fig. 7. In the seventh group (Infected/methanolic extract of *Feijoa* fruit), an infiltration of inflammatory cells was observed with an increase in goblet cell and enterocyte activity, Fig. 8. Regarding the eighth group (Infected/Antibiotic), inflammation and hyperemia as well as increased of goblet cell activity was observed, Fig. 9.

## Discussion

*Salmonella,* one of the most important members of the *Enterobacterales *family is widely known as one of the main causes of infectious diarrheal diseases. *Salmonella* enterica serovars Typhi, Paratyphi A, Paratyphi B and Paratyphi C are referred to as typhoid *Salmonella*, whereas other serovars are categorized as non-typhoid *Salmonella* (NTS). Typhoidal *Salmonella* strains are primarily restricted to the human host and are responsible for causing typhoid and paratyphoid fever, collectively referred to as enteric fever. On the other hand, NTS strains may have a broader host range, and infect or colonize various vertebrates, or they may be adapted to or restricted to certain non-human animal species^26^.

The treatment of infections caused by *Salmonella* can be a difficult undertaking. The emergence of serovars that are resistant to multiple drugs is a key factor in the ineffectiveness of treatment for human infections, emphasizing the need to consider antimicrobial options as a means of control. On the other hand, antibiotics are sometimes associated with opposite side effects such as hypersensitivity and allergic reactions^27^. Therefore, the identification of new sources of antimicrobial agents is important for the treatment of infectious diseases caused by bacterial pathogens. Due to their antimicrobial characteristics, extracts and nanoparticles have the ability to serve as viable alternatives to certain conventional antibiotics in the treatment of infections caused by bacterial pathogens, such as *Salmonella*^28,29^. The main aim of the present study was to investigate the antibacterial effect of methanolic extract of *Feijoa* fruit and FF-AgNPs on *Se*sT, as it is important to utilize the above mentioned agents with antibacterial properties.

In the present study, the antibacterial activity of methanolic extract of *Feijoa* fruit and its nanosilver (FF-AgNPs) against *Se*sT ATCC 14,028 was tested. Here, we used conventional antimicrobial treatment against an ATCC strain (with the antibiotic ceftriaxone) to illustrate the established paradigm of antimicrobial therapy. Subsequently, we demonstrated the effectiveness of two potential candidates with antimicrobial properties. The results of the MIC and MBC tests demonstrate that these agents have considerable antibacterial activity against *Se*sT and their efficacy is comparable to that of ceftriaxone. In accordance with the findings of Manilal et al., the utilization of plant extracts is considered to have bactericidal properties when the ratio of MBC to MIC is less than or equal to 2. Conversely, the extract is considered bacteriostatic if this ratio exceeds 2. However, a ratio equal to or greater than 16 indicates a lack of efficacy of the extract^30^, Since the ratio of MBC to MIC for FF-AgNPs and *Feijoa* fruit extract is 2 and 0.5, respectively, it can be concluded that these agents exhibit a bactericidal effect.

In addition to performing in-vitro analysis, our in-vivo studies have demonstrated the beneficial effects of the use of *Feijoa* fruit extract and FF-AgNPs, both in surveillance of infected mice and in evaluating the histopathological results. According to the current results, *Feijoa* fruit extract and FF-AgNPs significantly prevent the death of infected mice and reduce the growth of *Se*sT. Moreover, these antimicrobial agents have the potential to reduce weight loss in a similar manner to the antibiotic treatment, effectively minimizing it. The histopathology results also revealed the advantageous impacts of *Feijoa* fruit extract and FF-AgNPs in enhancing the condition of the gut. These two antimicrobial agents, similar to the ceftriaxone, have the capability to significantly reduce bleeding and inflammation.

Another notable outcome of the present study was the presence of an inflammatory state evident in the non-infected mice subjected to antibiotic treatment, wherein a notable infiltration of inflammatory cells was observed. Conversely, only mild inflammation was evident in the non-infected mice treated with *Feijoa* fruit extract and FF-AgNPs. This observation could potentially suggest, as Scott et al. similarly documented, that antibiotic administration may contribute to a predisposition to intestinal inflammation^31^.

Regarding intestinal conditions, our results have shown that FF-AgNPs has higher intestinal activity and better regulation of infectious agents and higher intestinal absorption capacity compared to *Feijoa*. Conversely, *Feijoa* fruit extract exhibits a higher absorption rate in the intestine but at the same time leads to increased goblet cell activity. This phenomenon leads to a decrease in intestinal motility due to the protective role of the mucus on the epithelial surface of the colon against various stimuli, possibly due to the intestinal response to *Feijoa* extract as a stimulant. While antibiotics have the function of suppressing infectious agents, they also have the effect of reducing the activity of the absorbing enterocytes. Our results suggest that all three antimicrobial agents, including *Feijoa* fruit extract*,* FF-AgNPs and ceftriaxone could have a significant effect on improving the condition of the infected mice. However, since antibiotics could have various side effects, it is preferable to use the agents with the least side effects.

A limitation of this study is the absence of systemic blood biomarkers (e.g., IL-6, TNF-α, or CRP) to assess inflammatory responses, as the current work focused primarily on histopathological analysis of intestinal tissues to evaluate local anti-infective effects. In future studies, the measurement of relevant systemic inflammatory markers in the blood will be included to provide a more comprehensive understanding of the inflammatory status. Additionally, the synergistic potential of *Feijoa sellowiana* methanolic extract and nanosilver in combination with antibiotic could be explored using the checkerboard assay to calculate the Fractional Inhibitory Concentration (FIC) index, allowing identification of optimal therapeutic concentrations and interactions against *Salmonella* infections.

## Conclusions

In conclusion, the results of this study highlight the significant antibacterial activity of both methanolic extract and nanosilver of *Feijoa* fruit against *Se*sT in BALB/c mice. The results show that these extracts not only have a strong bactericidal effect, as demonstrated by the minimal inhibitory concentration and bactericidal concentration, but also contribute to an improved survival rate in infected mice compared to untreated controls. Given the increasing challenge of antibiotic resistance, the use of *Feijoa* and its nano-silver extract represents a promising alternative or complementary strategy for the treatment of bacterial infections. These results support further investigation of the therapeutic potential of *Feijoa* as an effective adjunct to antibiotic treatment, which could be a valuable tool in the fight against resistant microbial pathogens.

## Data Availability

All data generated or analyzed during this study are included in this published article.
